# The value of haematological parameters and serum tumour markers for predicting KRAS mutations in 784 Chinese colorectal cancer patients: a retrospective analysis

**DOI:** 10.1186/s12885-020-07551-4

**Published:** 2020-11-12

**Authors:** Yinghao Cao, Junnan Gu, Lizhao Yan, Shenghe Deng, Fuwei Mao, Wentai Cai, Hang Li, Xinghua Liu, Jiliang Wang, Ke Wu, Kailin Cai

**Affiliations:** 1 Department of Gastrointestinal Surgery, Union Hospital, Tongji Medical College, Huazhong University of Science and Technology, Wuhan, 430022 Hubei China; 2 College of life Science and Technology, Huazhong University of Science and Technology, Wuhan, 430022 Hubei China

**Keywords:** Haematological parameters, Serum tumour markers, Colorectal cancer, KRAS mutation

## Abstract

**Background:**

Identifying the mutation status of KRAS is important for optimizing treatment in patients with colorectal cancer (CRC). The aim of this study was to investigate the predictive value of haematological parameters and serum tumour markers (STMs) for KRAS gene mutations.

**Methods:**

The clinical data of patients with colorectal cancer from January 2014 to December 2018 were retrospectively collected, and the associations between KRAS mutations and other indicators were analysed. Receiver operating characteristic (ROC) curve analysis was performed to quantify the predictive value of these factors. Univariate and multivariate logistic regression models were applied to identify predictors of KRAS mutations by calculating the odds ratios (ORs) and their corresponding 95% confidence intervals (CIs).

**Results:**

KRAS mutations were identified in 276 patients (35.2%). ROC analysis revealed that age, CA12–5, AFP, SCC, CA72–4, CA15–3, FERR, CYFRA21-1, MCHC, and tumor location could not predict KRAS mutations (*P* = 0.154, 0.177, 0.277, 0.350, 0.864, 0.941, 0.066, 0.279, 0.293, and 0.053 respectively), although CEA, CA19–9, NSE and haematological parameter values showed significant predictive value (*P* = 0.001, < 0.001, 0.043 and *P* = 0.003, < 0.001, 0.001, 0.031, 0.030, 0.016, 0.015, 0.019, and 0.006, respectively) but without large areas under the curve. Multivariate logistic regression analysis showed that CA19–9 was significantly associated with KRAS mutations and was the only independent predictor of KRAS positivity (*P* = 0.016).

**Conclusions:**

Haematological parameters and STMs were related to KRAS mutation status, and CA19–9 was an independent predictive factor for KRAS gene mutations. The combination of these clinical factors can improve the ability to identify KRAS mutations in CRC patients.

## Background

Colorectal cancer (CRC) is one of the most common malignant diseases and is the third most common cancer and the third leading cause of mortality in America [1], and its incidence and mortality are ranked fifth in China [2]. Despite advances in both prevention and treatment, metastatic colorectal cancer (mCRC) remains the second-leading cause of cancer-related mortality in the United States [3]. The discovery of mutant KRAS as a predictor of resistance to epidermal growth factor receptor (EGFR) monoclonal antibodies led to a major change in the treatment of metastatic colorectal cancer [4]. The determination of molecular markers (KRAS and BRAF oncogenes) has been used to stratify cases of colorectal cancer, and the choice of treatment and advances in targeted therapy have yielded significant increases in patient survival.

KRAS is an important effector of ligand-bound EGFR, and KRAS signally is mainly but not exclusively through BRAF and the MAPK axis. Approximately 32–40% of colorectal cancers harbour a KRAS mutation. Approximately 85–90% of these mutations occur in codons 12 or 13. The remaining mutations mainly occur in codons 61 (5%) and 146 (5%). These mutations disable GTPase activity, causing tumour-associated KRAS to accumulate in the active GTP-bound conformation [5, 6]. It has been demonstrated that anti-EGFR antibody treatment with cetuximab and with panitumumab did not confer benefits for tumours with a mutant KRAS gene [7, 8]. The guidelines of the National Comprehensive Cancer Network recommend that the tumour tissues of all patients with suspected or proven metastatic CRC should undergo genotyping for KRAS mutations [9]. Therefore, identifying the KRAS mutation status of CRC, either before the application of anti-EGFR treatment or during treatment, is required to predict the therapeutic effect and determine individual treatment strategies. Although pathologic analyses of KRAS mutation status are regarded as the gold standard in current clinical practice, these tests are sometimes not feasible (poor specimen quality and expensive testing) [10]. Therefore, there is an urgent need to develop a low-cost, simple and non-invasive detection method.

At present, serum tumour markers (STMs) and haematological parameters play important roles in the diagnosis, follow-up, evaluation of treatment response and prediction of recurrence of some cancers [11]. Previous research indicated that STMs (CEA, CA-125, SCC, NSE, and CYFRA21-1) are the best tumour markers for CRC patients [12, 13]. Some authors suggest that some haematological parameters can be inflammation markers and are accepted as important prognostic indicators of various malignancies. These parameters have been increasingly used in colorectal cancer patients [14–16]. Therefore, we hypothesized that a nonpathological method with the ability to predict the KRAS mutation status of CRC would enable precision medicine. In this study, we aimed to investigate whether haematological parameters and STMs could be used to predict the KRAS mutation status of CRC.

## Methods

### Study design and patient cohort

From January 2014 to December 2018, 841 patients with CRC visited Wuhan Union Medical College Hospital. We retrospectively collected the demographic data, haematological parameters, STMs and KRAS status of the patients. The study was approved by the institutional review board for human investigation (national software copyright 2019SR1267841). The haematological parameters included WBC, MON, MLR, HCT, HGB, AVEMPV, MCH, MCHC, and HDLC, and the serum tumour markers included CEA, SCC, CYFRA 21-1, NSE, AFP, CA125, CA 19–9, CA 15–3, FERR and CA 72–4.

- A total of 841 patients were identified, and 57 patients who met the following criteria were excluded from the study: (1) treatment before KRAS status detection (35 patients); (2) history of tumours (14 patients); and (3) severe cardiovascular disease (8 patients).

### Haematological parameters and STM measurements

Haematological parameters were detected before detecting KRAS mutation status, and STMs were detected by a commercial chemiluminescence immunoassay kit (Abbott Laboratories, I4000, America). After admission, blood samples were obtained from all participants by peripheral venocentesis before any anticancer treatment was administered, and the KRAS mutation status was detected after surgery or biopsy after an interval of approximately 2 weeks.

### KRAS mutation analysis

Preoperative biopsy or postoperative tumour specimens were used for KRAS gene detection. Tumour tissues were fixed in 10% neutral buffered formalin, processed, and then embedded in paraffin for light microscopy. The sections were stained with haematoxylin and eosin (H&E) for histological examination. The Cobas DNA sample preparation kit was used to extract DNA from formalin-fixed paraffin-embedded tissue sections (Roche Molecular Systems, Inc., Branchburg, NJ, USA) according to the instructions, and the reaction was carried out with the Mx3000PTM real-time PCR system (Stratagene, La Jolla, USA). Using a real-time polymerase chain reaction assay, the Cobas KRAS Mutation Test (Roche Molecular Systems, Inc.) and LightMix KRAS and NRAS kits (Roche Molecular Systems, Inc.) were applied to detect KRAS mutations. Tumours harbouring KRAS mutations in either preoperative biopsy or post-treatment resection specimens were considered KRAS mutants.

### Statistical analysis

Parametric tests (independent samples t-test) were applied to data with a normal distribution, and nonparametric tests (Mann–Whiney U-test) were applied to data with non-normally distributions. The relationships among haematological parameters, STM levels and gene mutations were analysed using univariate logistic regression. The significant indexes in the single-factor analysis and the indexes that influenced the gene mutation status of the patients were selected for multivariate analysis. The data are expressed as the mean ± SD or median (interquartile range), as appropriate. Different predictive models were compared based on areas under the curve (AUCs). All statistical analyses were performed using SAS 9.4 (SAS Institute Inc., Cary, North Carolina, USA) and R3.5.1 (R Foundation for Statistical Computing, Vienna, Austria), with a two-sided *P* < 0.05 considered statistically significant.

## Results

### Patient clinical characteristics

**Fig. 1:**
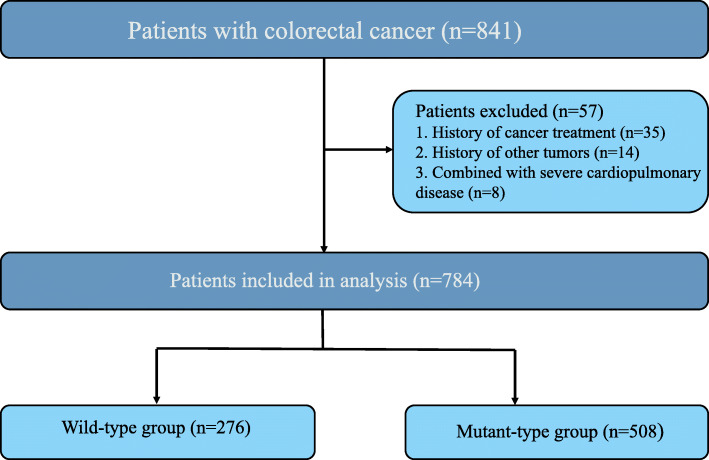
Study design and algorithm of patient selection

**Fig. 2:**
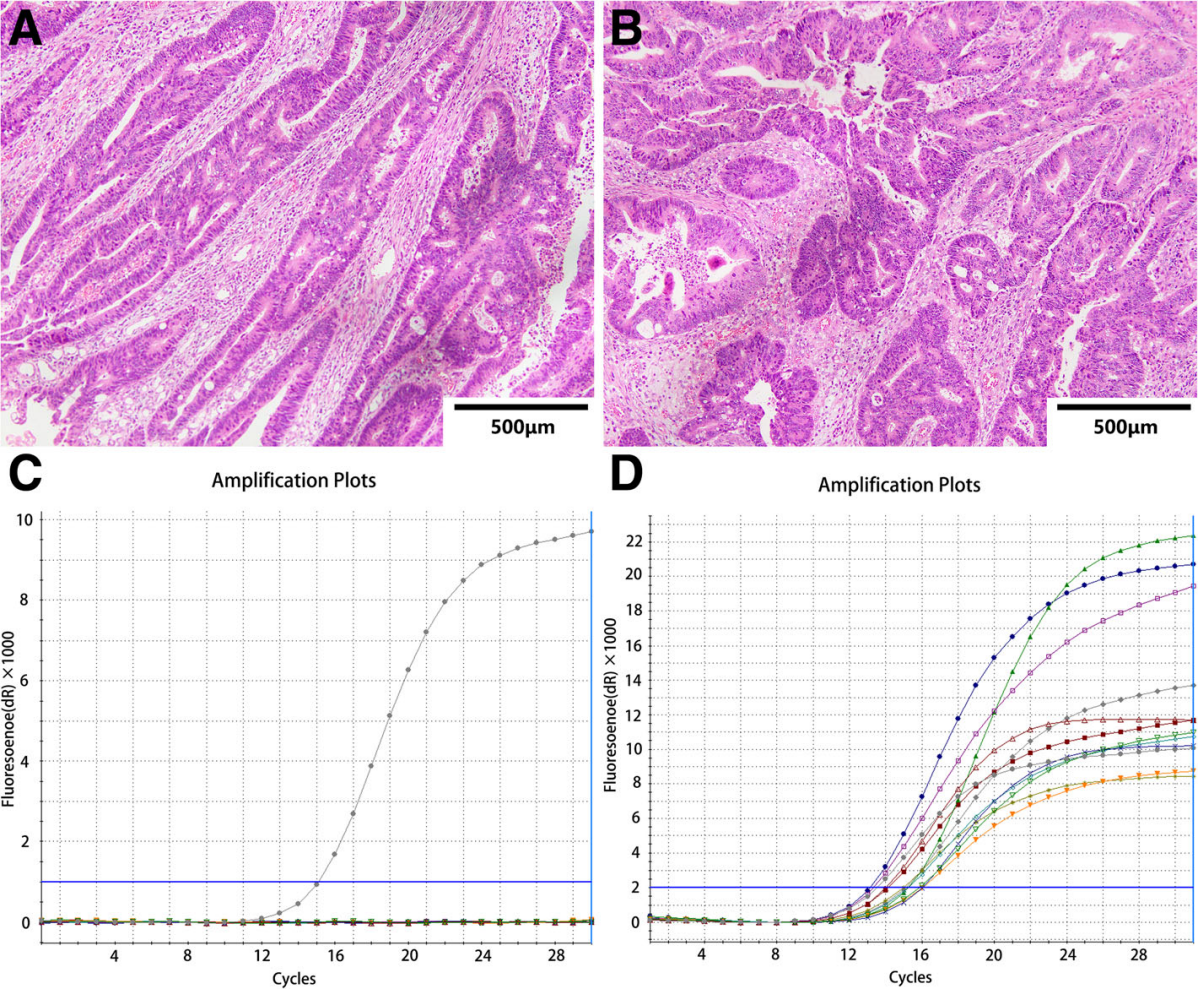
Representative histological images with KRAS wild-type CRC patient. Top panel, findings of a 79-year-old man with KRAS wild-type CRC (**a**, **b**). Bottom panel, with hematoxylin-eosin staining showing histological type and the ARMS method, (**c**, **d**) demonstrates the KRAS status

**Fig. 3:**
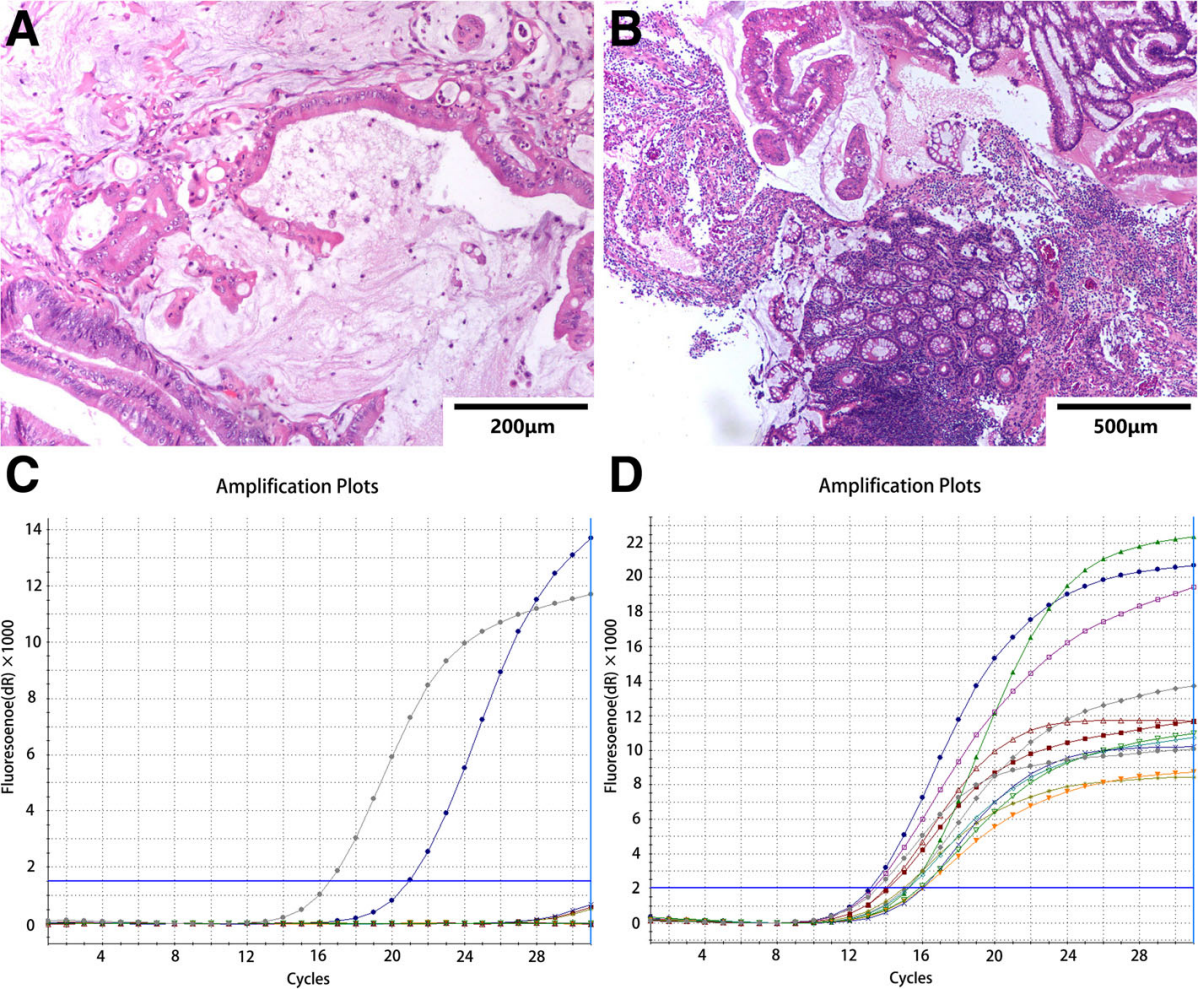
Representative histological images with KRAS mutant-type CRC patient. Top panel, findings of a 54-year-old man with KRAS mutant-type CRC (**a**, **b**). Bottom panel, with hematoxylin-eosin staining showing histological type and the ARMS method, (**c**, **d**) demonstrates the KRAS status

Among the 784 CRC patients whose KRAS status was tested in our hospital between January 2014 and December 2018, 473 were male, and 311 were female. The mean age of the patients was 57.12 ± 12.12 years (range, 29–85). In 276 cases (35.2%), mutations in the KRAS gene were detected, while in 508 cases, mutations were not observed (wild-type KRAS) (Fig. 1). Typical histological images of four patients with CRC with mutant or wild-type KRAS are shown in Figs. 2 and 3.

**Table 1:** Mean, standard deviation, minimum and maximum values of hematological parameters STMs

	N	mean	Std.dev	25%th	Median	75%th	Min	Max
Age	784	57.12	12.12	49.00	58.00	66.00	17.00	89.00
CEA	784	35.29	154.54	2.20	4.05	11.22	0.20	1500
CA125	784	25.13	85.72	8.30	11.85	19.12	2.30	1972.50
CA19–9	784	96.99	455.89	4.10	9.30	27.10	1.50	10,990.60
AFP	720	5.00	53.34	1.90	2.60	3.40	0.70	1430.10
SCC	547	0.92	1.42	0.50	0.70	1.00	0.10	31.30
NSE	698	17.87	15.49	13.11	15.79	19.25	7.32	370.00
CA72–4	698	14.15	43.19	1.25	2.37	6.73	0.43	310.00
CA15–3	572	8.53	4.70	5.30	7.30	10.40	2.50	57.00
FERR	527	128.09	290.69	20.30	59.00	136.60	1.00	4268.60
CYFRA21-1	547	4.63	10.80	1.49	2.15	3.32	0.51	100
WBC	784	6.18	2.17	4.77	5.80	7.10	1.14	19.41
MON	784	0.44	0.18	0.32	0.41	0.53	0.05	1.67
MLR	784	0.31	0.17	0.20	0.28	0.37	0.06	1.51
Mon%	784	7.37	2.53	5.70	7.10	8.70	1.10	22.20
HCT	784	35.74	6.43	31.40	36.80	40.40	16.10	51.90
HGB	784	116.73	24.42	101	1210	134	32.90	182.00
AVEMPV	645	13.84	2.66	11.70	13.80	16.10	8.10	23.80
MCH	784	27.95	3.93	26.10	29.20	30.50	12.80	36.80
MCHC	784	325.12	16.24	318.00	329.00	336.00	233.00	364.00
MCV	784	85.65	9.26	81.60	88.30	91.70	54.70	110.10
HDLC	689	1.14	0.32	0.92	1.11	1.33	0.15	2.74

*CEA* Carcinoembryonic antigen, *CA 125* Carbohydrate antigen 125, *CA 19–9* Carbohydrate antigen 19–9, *SCC* Squamous cell carcinoma antigen, *NSE* Neuronspecific enolase, *CA 724* Carbohydrate antigen 72–4, *FERR* Ferritin, *CYFRA21-1* Human cytokeratin fragment antigen 21–1, *WBC* White blood cell, *MON* Monocyte, *MLR* Monocyte /Lymphocyte, *HCT* Hematocrit, *HGB* Hemoglobin, *AVEMPV* Mean platelet volume, *MCH* Mean corpuscular hemoglobin, *MCHC* Mean corpusular hemoglobin concerntration, *MCV* Mean corpuscular volume, *HDLC* High-density lipoprotein

Primary tumours were observed in the ascending colon (*n* = 205), transverse colon (*n* = 40), descending colon (*n* = 50), sigmoid colon (*n* = 145) and rectum (*n* = 344). The mean values of the haematological parameters and STMs in these patients are shown in Table 1.

**Table 2:** Differences in values of hematological parameters and STMs between the wild-type and mutant KRAS groups

	KRAS Wild-type			KRAS Mutantion			*P*
	N	Median	Std.dev	N	Median	Std.dev	
Age	508	58.00	12.54	276	58.00	11.29	0.154
Tumor location	508			276			0.019
Ascending colon	115 (14.7)^a^	–	–	91 (11.6)	–	–	
Transverse colon	27 (3.4)	–	–	12 (1.5)	–	–	
Descending colon	32 (4.1)	–	–	17 (2.2)	–	–	
Sigmoid	106 (13.5)	–	–	40 (5.1)	–	–	
Rectum	228 (29.1)	–	–	116 (14.8)	–	–	
CEA	508	3.70	124.99	276	4.80	196.67	< 0.001
CA125	508	12.25	103.17	276	11.20	35.23	0.175
CA199	508	8.00	220.29	276	12.40	706.28	< 0.001
AFP	455	2.60	66.93	265	2.60	6.52	0.277
SCC	358	0.70	1.72	189	0.70	0.49	0.348
NSE	435	16.01	8.26	263	15.27	22.93	0.043
CA72.4	435	2.38	41.83	263	2.34	45.43	0.863
CA15.3	378	7.30	4.99	194	7.25	4.11	0.941
FERR	341	63.80	301.84	186	48.25	269.67	0.066
CYFRA21.1	358	2.12	10.42	189	2.25	11.52	0.279
WBC	508	5.93	2.19	276	5.59	2.13	0.003
MON	508	0.43	0.19	276	0.38	0.16	0.846
MLR	508	0.29	0.19	276	0.25	0.12	< 0.001
Mon%	508	7.29	2.60	276	6.78	2.36	0.005
HCT	508	37.05	6.28	276	36.55	6.64	0.031
HGB	508	123.00	23.54	276	119.00	25.73	0.030
AVEMPV	423	13.90	2.67	222	13.50	2.61	0.016
MCH	508	29.30	3.77	276	29.00	4.18	0.015
MCHC	508	328.00	14.98	276	329.00	18.25	0.292
MCV	508	88.60	8.97	276	87.20	9.68	0.019
HDLC	443	1.08	0.33	246	1.16	0.31	0.006

*CEA* Carcinoembryonic antigen, *CA 125* Carbohydrate antigen 125, *CA 19–9* Carbohydrate antigen 19–9, *SCC* Squamous cell carcinoma antigen, *NSE* Neuronspecific enolase, *CA 724* Carbohydrate antigen 72–4, *FERR* Ferritin, *CYFRA21-1* Human cytokeratin fragment antigen 21–1, *WBC* White blood cell, *MON* Monocyte, *MLR* Monocyte /Lymphocyte, *HCT* Hematocrit, *HGB* Hemoglobin, *AVEMPV* Mean platelet volume, *MCH* Mean corpuscular hemoglobin, *MCHC* Mean corpusular hemoglobin concerntration, *MCV* Mean corpuscular volume, *HDLC* High-density lipoprotein

^a^The percentage in brackets represents the percentage of the total number of patients

Analyses using the Mann-Whitney U test showed that there were no significant differences between the wild-type and mutant KRAS groups in terms of age, CA12–5, AFP, SCC, CA72–4, CA15–3, FERR, CYFRA21.1, MON, and MCHC values (*P* > 0.05). The WBC, MLR, Mon%, HCT, HGB, AVEMPV, MCH, MCV, and HDLC values were significantly lower in the mutant group (*P* < 0.05), and CEA, CA19–9, and NSE values were significantly higher in the mutant group(*P* < 0.05) (Table 2). Furthermore, no significant difference was observed between males and females in terms of KRAS mutation according to the results of the Pearson chi-square test (*P* = 0.430).

### Predictive model analysis

The predictive power of haematological parameters and STMs for mutations in the KRAS oncogene was evaluated with ROC curves. Areas under the ROC curve are shown in Table 3. From the ROC analyses, significant *P* values were obtained for CEA, CA19–9, NSE, WBC, MON, MLR, Mon%, HCT, HGB, AVEMPV, MCH, MCV, and HDLC (*P* < 0.05). However, these parameters did not have very high AUC values, and MON had the highest AUC (0.606). In multivariate logistic regression analysis, the predictive power of age, haematological parameters and STMs for KRAS gene mutations was evaluated. The *P* values and OR values are summarized in Table 4. The only significant association was observed between CA19–9 and KRAS mutations.

**Table 3:** Shows ROC analysis, AUC (area under curve), standard error, condence interval and *P* values of hematological parameters and STMs

	AUC	Standard error	95%CI		*P*
			Low bound	Upper bound	
Age	0.531	0.021	0.489	0.572	0.154
CEA	0.574	0.022	0.531	0.617	0.001
CA125	0.529	0.021	0.487	0.571	0.177
CA199	0.579	0.021	0.537	0.620	< 0.001
AFP	0.524	0.023	0.480	0.569	0.277
SCC	0.524	0.026	0.473	0.575	0.350
NSE	0.546	0.023	0.501	0.590	0.043
CA72.4	0.504	0.022	0.460	0.548	0.864
CA15.3	0.502	0.025	0.452	0.552	0.941
FERR	0.549	0.026	0.497	0.600	0.066
CYFRA21-1	0.528	0.026	0.477	0.579	0.279
WBC	0.563	0.021	0.521	0.605	0.003
MON	0.606	0.021	0.565	0.648	< 0.001
MLR	0.571	0.021	0.531	0.612	0.001
Mon%	0.561	0.021	0.519	0.602	0.005
HCT	0.546	0.022	0.504	0.589	0.031
HGB	0.547	0.022	0.504	0.589	0.030
AVEMPV	0.557	0.024	0.511	0.604	0.016
MCH	0.552	0.021	0.510	0.594	0.015
MCHC	0.523	0.022	0.480	0.566	0.293
MCV	0.551	0.021	0.509	0.593	0.019
HDLC	0.563	0.023	0.519	0.607	0.006
Tumor location	0.542	0.022	0.499	0.585	0.053

*CEA* Carcinoembryonic antigen, *CA 125* Carbohydrate antigen 125, *CA 19–9* Carbohydrate antigen 19–9, *SCC* Squamous cell carcinoma antigen, *NSE* Neuronspecific enolase, *CA 724* Carbohydrate antigen 72–4, *FERR* Ferritin, *CYFRA21-1* Human cytokeratin fragment antigen 21–1, *WBC* White blood cell, *MON* Monocyte, *MLR* Monocyte /Lymphocyte, *HCT* Hematocrit, *HGB* Hemoglobin, *AVEMPV* Mean platelet volume, *MCH* Mean corpuscular hemoglobin, *MCHC* Mean corpusular hemoglobin concerntration, *MCV* Mean corpuscular volume, *HDLC* High-density lipoprotein

**Table 4:** Multivariate logistic regression analysis for the prediction of KRAS gene mutations

	Univariate *P* value	Multivariate *P* value	OR	95%CI for OR	
				Lower	Upper
Age	0.156	0.417	1.008	0.989	1.029
CEA	0.023	0.907	1.000	0.998	1.002
CA125	0.161	0.062	0.992	0.983	0.999
CA199	0.055	0.016	1.001	1.0002	1.002
AFP	0.595	0.708	0.999	NA	1.002
SCC	0.248	0.162	0.755	0.497	1.035
NSE	0.793	0.946	1.001	0.983	1.027
CA72.4	0.922	0.042	0.992	0.983	0.999
CA15.3	0.733	0.512	0.982	0.928	1.036
FERR	0.653	0.368	1.000	0.999	1.002
CYFRA21.1	0.535	0.311	1.018	0.982	1.056
WBC	0.017	0.948	1.009	0.773	1.309
MON	0.000	0.366	0.200	0.006	6.93
MLR	0.000	0.666	0.589	0.050	6.278
Mon%	0.002	0.997	1.000	0.792	1.265
HCT	0.023	0.176	1.492	0.829	2.757
HGB	0.012	0.158	0.880	0.729	1.054
AVEMPV	0.018	0.756	0.985	0.895	1.083
MCH	0.011	0.605	1.553	0.288	8.339
MCHC	0.034	0.840	1.012	0.900	1.141
MCV	0.012	0.559	0.853	0.497	1.461
HDLC	0.031	0.116	1.746	0.872	3.521
Tumor location					
Ascending colon	ref	–	–	–	–
Transverse colon	0.123	0.026	0.295	0.094	0.832
Descending colon	0.229	0.274	0.582	0.216	1.518
Sigmoid	0.001	0.006	0.363	0.173	0.744
Rectum	0.015	< 0.001	0.286	0.152	0.53

*CEA* Carcinoembryonic antigen, *CA 125* Carbohydrate antigen 125, *CA 19–9* Carbohydrate antigen 19–9, *SCC* Squamous cell carcinoma antigen, *NSE* Neuronspecific enolase, *CA 724* Carbohydrate antigen 72–4, *FERR* Ferritin, *CYFRA21-1* Human cytokeratin fragment antigen 21–1, *WBC* White blood cell, *MON* Monocyte, *MLR* Monocyte /Lymphocyte, *HCT* Hematocrit, *HGB* Hemoglobin, *AVEMPV* Mean platelet volume, *MCH* Mean corpuscular hemoglobin, *MCHC* Mean corpusular hemoglobin concerntration, *MCV* Mean corpuscular volume, *HDLC* High-density lipoprotein

## Discussion

Malignant neoplasms are an increasing medical problem worldwide, and CRC is among the top 10 causes of mortality. KRAS mutations occur at a late stage in adenoma development and are a key element for mCRC development. These mutations are found in 30 to 50% of all tumours, especially in codon 12 (80% of reported mutations) and codon 13 (20%) [17]. Zy Chen et al. performed a study on 342 colorectal cancer patients and detected KRAS mutations in 52.6% of the patients [18]. In our study, KRAS mutations were detected in 276 cases (35.2%), which is consistent with the results of previous studies. Furthermore, the patients with KRAS mutations in our study were predominantly female, although this difference was not significant.

A series of studies have reported that anti-EGFR monoclonal antibody therapy was associated with improvements in both prognosis and compliance, as well as reductions in toxicity and side effects, and patients with wild-type KRAS metastatic CRC who received anti-EGFR monoclonal antibody therapy (cetuximab) and the FOLFIRI regimen (folinic acid, 5-fluorouracil, irinotecan) experienced prolonged survival up to 33.1 months [19, 20]. Some authors found that up to 50–65% of patients with wild-type KRAS tumours were resistant to EGFR monoclonal antibodies [6]. Therefore, the confirmation of KRAS status is important for optimizing treatments in patients with CRC.

Currently, the gold standard for KRAS mutation detection is conventional PCR amplification followed by direct sequencing. However, in clinical practice, genetic analysis is not available in some centres, and it is sometimes difficult to obtain adequate tumour tissues for genetic testing. Previous studies have shown that 18F-FDG uptake on PET/CT was associated with KRAS mutation status and could be combined with other factors to detect KRAS mutation status [21]. However, due to the shortcomings of a large dose of radiation and high price, the use of this PET/CT has been greatly limited. Therefore, a non-invasive and easy-to-use method is needed to predict KRAS mutation status, especially in CRC patients in China.

Very few studies in the medical literature have evaluated the correlation between haematological parameters and KRAS mutations. The study performed by Chen et al. 2014 found no significant correlation between NLR and KRAS mutation (OR: 0.98; 95% CI: 0.571.69; *P* = 1.000). Ali Ozan Oner et al. also found that a significant correlation did not exist between KRAS and NLR [22]. However, in our study, a significant correlation did exist between haematological parameters (WBC, MLR, Mon%, HCT, HGB, AVEMPV, MCH, MCV) and KRAS mutation, and there was also a significant difference in CEA, CA19–9, and NSE values between patients with wild-type and mutant KRAS *(P* < 0.05). This result is contrary to previous results, mainly because of the large number of patients we included. Some studies have demonstrated the prognostic value of NLR and PLR in CRC patients [23, 24]. However, the use of other haematological parameters to evaluate KRAS gene mutation status has not been assessed. In our study, we found that haematological parameters (WBC, MON, MLR, HCT, HGB, AVEMPV, MCH, MCHC) were significantly correlated with KRAS gene mutations, and the values of these haematological parameters were lower in the mutant group than in the wild-type group.

Tumour markers have been used to monitor, diagnosis, stage, evaluate and determine recurrence [25, 26]. Selcukbiricik et al. investigated 215 patients with colorectal cancer, and they observed a significant difference in CEA values between patients carrying the mutant KRAS gene and those with the wild-type gene (*P* = 0.02). Li et al. [27] investigated 945 patients and observed a significant association of KRAS mutations with CEA and CA19–9 (*P* = 0.0001), which was similar to the finding in our study, and we found that CEA, CA19–9, and NSE were higher in the mutant group (*P* < 0.05). In our study, when ROC curves for CEA, CA19–9, and NSE were drawn based on KRAS mutation status, we obtained significant *P* values (*P* = 0.01, *P* < 0.001, and *P* = 0.043, respectively) for these parameters, but the AUCs (0.574, 0.579, and 0.546, respectively) were not very high. In multivariate logistic regression analysis, the predictive power of age, haematological parameters and STMs for KRAS gene mutations was evaluated. The only significant association was observed between CA19–9 and KRAS mutations (*P* = 0.029). This was a new finding that is different from previous studies and contributes to research in this field. We look forward to more patients being enrolled for subsequent analyses.

There are also some limitations to our study. First, as a retrospective study, information about the histopathological subtypes and pathological stages of colorectal cancers of some patients could not be obtained. Therefore, we did not divide and evaluate patients according to their histopathological subtypes and pathological stages, which would affect our results. Second, some haematological markers were missing and could potentially bias the results. Third, we did not evaluate treatment response according to haematological parameters or serum tumour marker levels, and we did not collect follow-up information after surgery and were unable to conduct a survival analysis, which may weaken the clinical significance of the study. However, we believe that the results of this study are accurate, as we had a large sample size. Therefore, our study was still representative, and we will design prospective studies to reduce the occurrence of bias in the following study.

## Conclusion

There were significant but not very strong associations of CEA, CA19–9, NSE, WBC, MON, MLR, Mon%, HCT, HGB, AVEMPV, MCH, and MCV with KRAS mutations, and CA19–9 was an independent predictive factor of KRAS gene mutations. The combination of these clinical factors can improve the ability to identify KRAS mutation status in CRC patients.

## Data Availability

The datasets used and/or analysed during the current study are available from the corresponding author on reasonable request.

## Abbreviations

AUC: Area under the curve
CA: Carbohydrate antigen
CEA: Carcinoembryonic antigen
CRC: Colorectal cancer
EGFR: Epidermal growth factor receptor
mCRC: Metastatic colorectal cancer
NSE: Neuron-specific enolase
ROC: Receiver operating characteristic
SCC: Squamous cell carcinoma
STMs: Serum tumour markers
CA 125: Carbohydrate antigen 125
CA 19–9: Carbohydrate antigen 19–9
CA 724: Carbohydrate antigen 72–4
FERR: Ferritin
CYFRA21-1: Cytokeratin fragment antigen 21–1
WBC: White blood cell
MON: Monocyte
MLR: Monocyte /Lymphocyte
HCT: Hematocrit
HGB: Hemoglobin
AVEMPV: Mean platelet volume
MCH: Mean corpuscular hemoglobin
MCHC: Mean corpusular hemoglobin concerntration
MCV: Mean corpuscular volume
HDLC: High-density lipoprotein
